# Supplementary material for: Fate mapping of peripherally-derived macrophages after traumatic brain injury in mice reveals a long-lasting population with a distinct transcriptomic signature
Source: Nat Commun. 2025 Oct 7;16:8898. doi: 10.1038/s41467-025-63952-8 (PMC12504527; doi:10.1038/s41467-025-63952-8)
Supplement: Supplementary file 1 — Supplementary Information [file 41467_2025_63952_MOESM1_ESM.pdf]

Supplementary Figure 1 - Blood and brain labeling at the time of injury

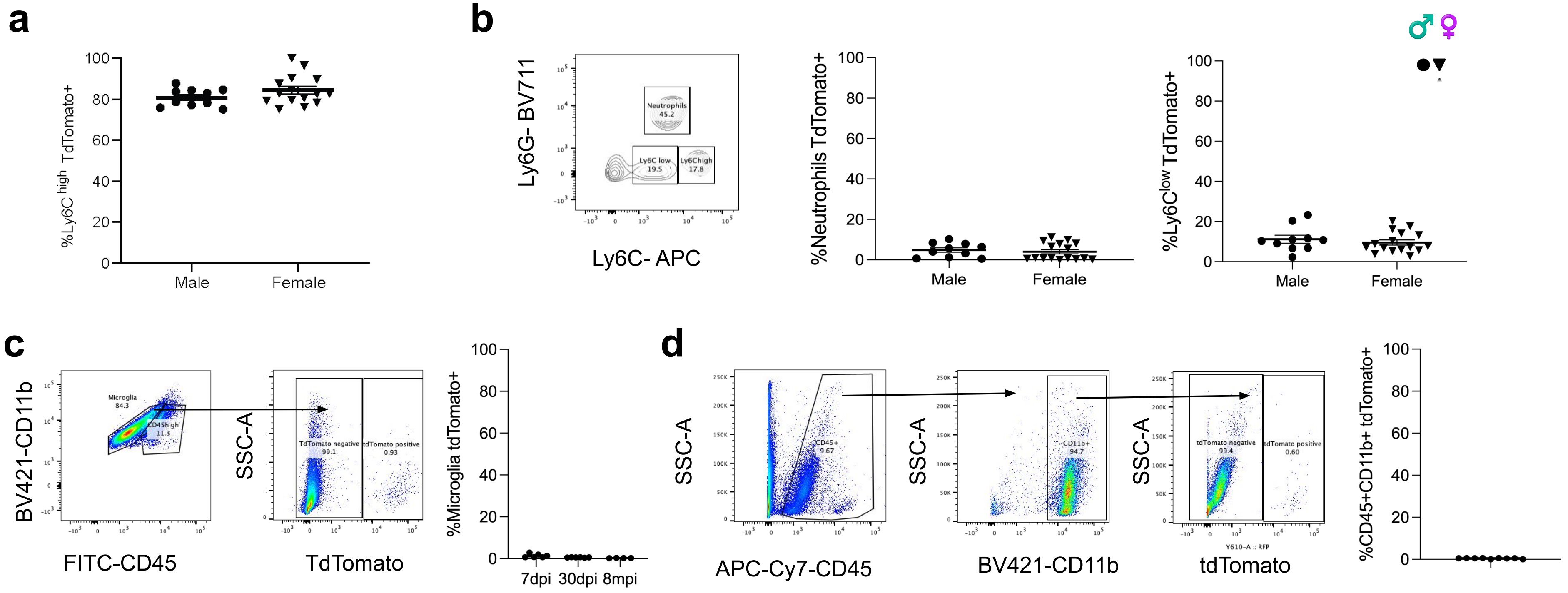

Supplementary Figure 1 -
(**a**) Off-target labeling of neutrophils (gated as CD11b+Ly6C+Ly6G+) and patrolling monocytes (gated as CD11b+Ly6G-Ly6Clow) after 3 doses of tamoxifen (at the time of injury) was below 15%. (**b**) Labeling efficiency expressed as percentage of blood Ly6C^{high} monocytes that were tdTomato+ at the day of injury (3 doses of tamoxifen, day 0). Individual animals are plotted (n = 10 male and 16 female mice). No sex differences were observed. (**c**) Off-target labeling of microglia (gated as CD11b+ CD45$^{mid/low}$) in Ccr2-creER^{T2}::Ai14D TBI mice. Labeling efficiency expressed as percentage of brain microglia that were tdTomato+. Individual animals are plotted (7dpi: n=6, 30dpi: n=6, 8mpi n=4). (**d**) No tdTomato+ events were detected in sham mice at 8mpi. Labeling efficiency expressed as percentage of brain CD45+CD11b+ cells that were tdTomato+. Individual animals are plotted (n=9). Bars are the mean of the examined variable ± SEM. Unpaired *t*-test. Source data are provided as a Source Data file.

Supplementary Figure 2 - No sex differences in cognitive deficits after TBI

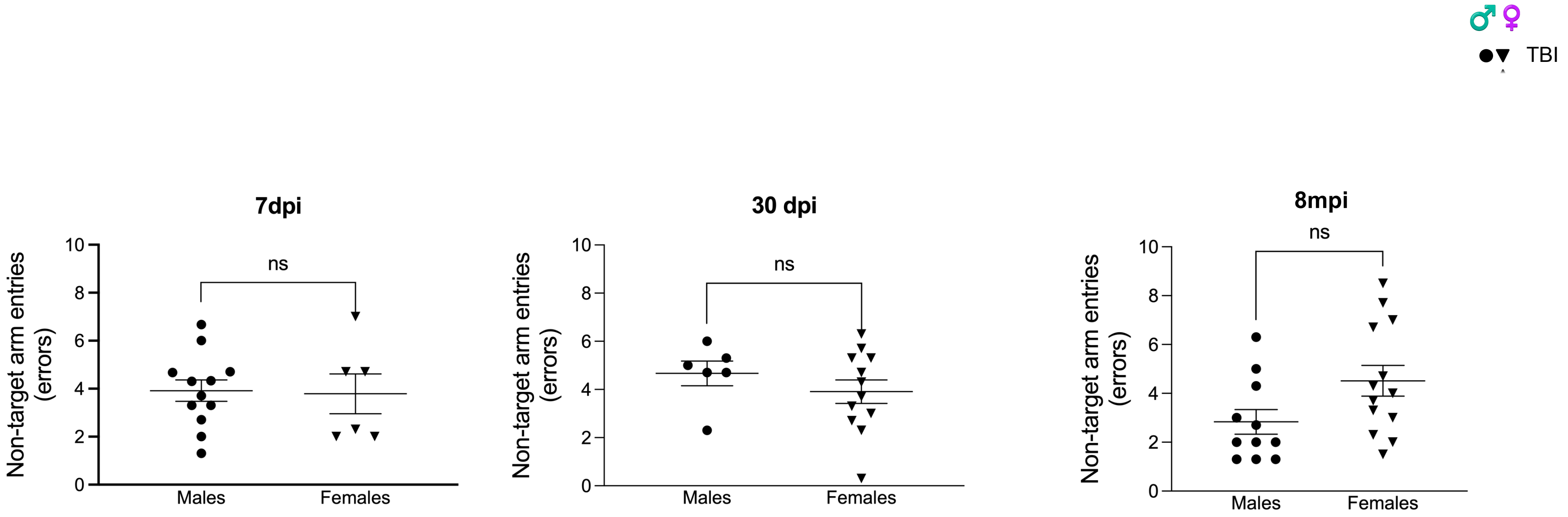

Supplementary Figure 2 -
Ccr2-creER^{T2}::Ai14D mice develop memory deficits after TBI. No sex differences were observed in the memory probe performance of TBI mice. Male mice are coded in circles and female in triangles. Individual animals are plotted (7dpi n = 12 males, 6 females, 30dpi n= 6males, 12 females, 8mpi n= 11 males, 13 females). Bars are the mean of the examined variable ± SEM. Unpaired *t*-test. Source data are provided as a Source Data file.

Supplementary Figure 3 - tdTomato+ colocalization with Iba1 and P2yr12 - quantification by coordinates

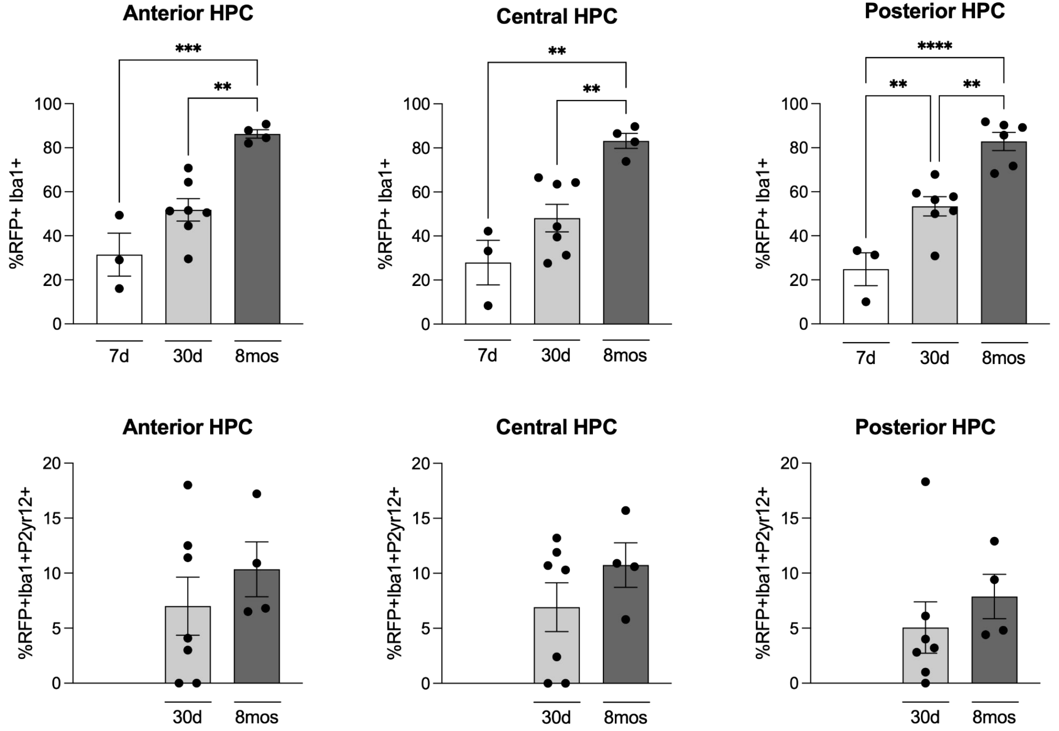

Supplementary Figure 3 -
For labeled macrophages visualization, pericontusional regions (top quarter of a coronal brain section) were acquired as 20x z-stacked tiled images. For each mouse, cavitation images were acquired at 3 different coordinates from Bregma: -1.34mm (Anterior), -1.81mm (Central) and -2.54mm (Posterior). Top: % of tdTomato+ cells that express Iba1+. Bottom: % of tdTomato+ cells that express Iba1 and P2yr12. Individual animals are plotted (7dpi n= 2 males, 1 female; 30dpi n=3 males, 4 females; 8mpi n=2 males, 2 females). Bars are the mean of the examined variable ± SEM. One-way ANOVA with Tuckey multiple comparisons test. Source data and statistical test results are provided as a Source Data file.

Supplementary Figure 4
tdTomato+ cells don't infiltrate the brain parenchyma of sham and don't migrate to the contralateral hemisphere of TBI Ccr2-creER^{T2}::Ai14D mice

a

7dpi **30dpi** **8mpi**

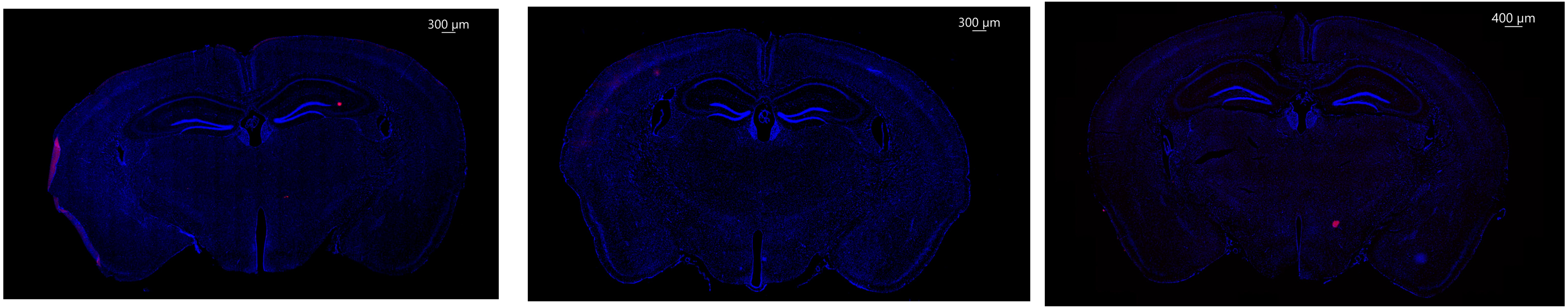

b

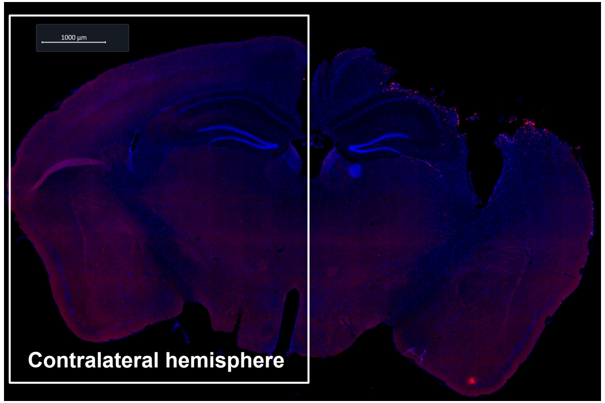

Supplementary Figure 4 -
a) No tdTomato+ cells were found in the coronal sections of sham Ccr2-creER^{T2}::Ai14D mice (**b**) tdTomato+ MDMs don't migrate to the contralateral hemisphere of TBI Ccr2-creER^{T2}::Ai14D mice at 7dpi. The experiment was independently replicated in two separate cohorts/time point with multiple biological replicates/cohort.

Supplementary Figure 5 - No sex differences in phagocytosis

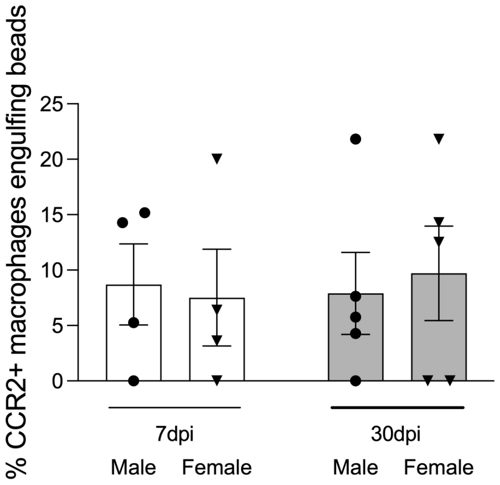

ANOVA table	SS (Type III)	DF	MS	F (DFn, DFd)	P value
Interaction	9.977	1	9.977	F (1, 14) = 0.1361	P=0.7178
Sex	0.4438	1	0.4438	F (1, 14) = 0.006052	P=0.9391
Time after injury	2.293	1	2.293	F (1, 14) = 0.03127	P=0.8622
Residual	1027	14	73.33		

Supplementary Figure 5 -
No sex differences were observed in the phagocytosis capability of TBI-induced infiltrated macrophage. Each dot represents the average of 2/4 measures/mouse.Individual animals are plotted (7dpi n= 4 males, 4 females; 30dpi n=5 males, 5 females). Data are expressed as the mean of the examined variable ± SEM. Two Way ANOVA. Source data are provided as a Source Data file.

Supplementary Figure 6 - Gating Strategy for RNAseq

a

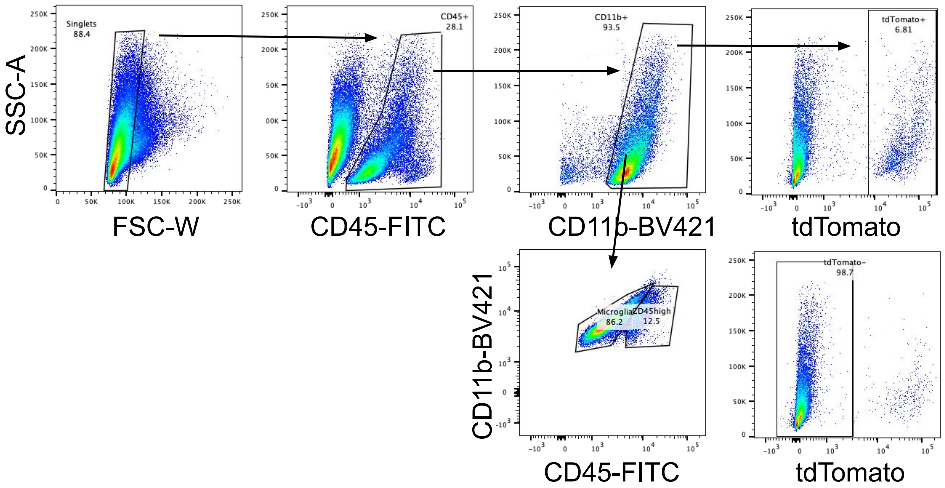

b

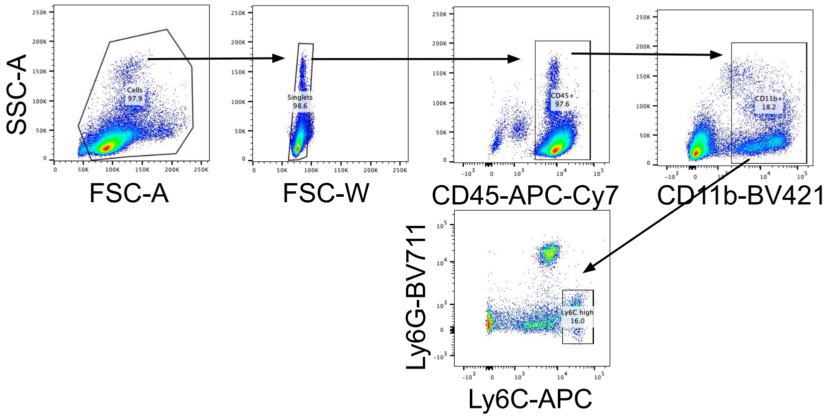

Supplementary Figure 6 - (a) Gating strategy for sorting MDMs`(CD45+, CD11b+, tdTomato+) and microglia (CD11b+, CD45$^{mid/low}$, tdTomato-) from whole brains. (**b**) Gating strategy for sorting inflammatory monocytes (CD45+, CD11b+, Ly6G-,Ly6C^{hi}) from blood samples.

Supplementary Figure 7 - Infiltrated macrophages (Ccr2) transcriptomes mapped onto ImmuneSigDB dataset

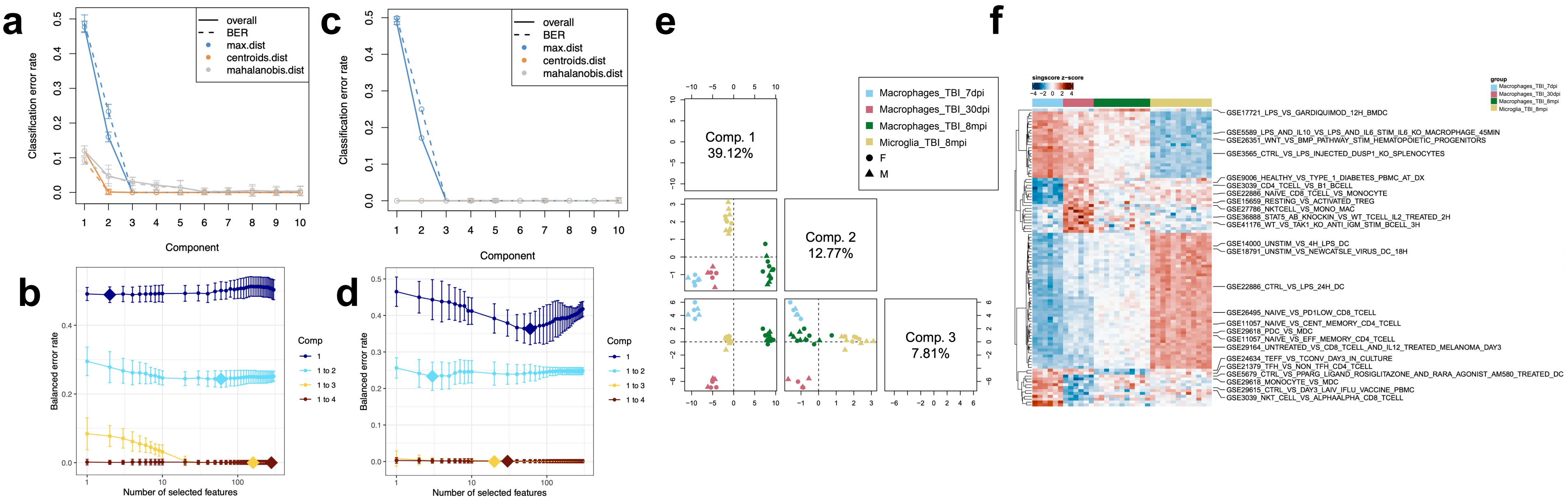

Supplementary Figure 7 - Classification error rates per number of sPLS-DA components for the (**a**) Reactome and (**c**) ImmuneSigDB databases. 3 components were selected for the final models for both databases. Balanced error rate per number of selected features per discriminant component for the (**b**) Reactome and (**d**) ImmuneSigDB databases. (**e**) Projections of samples onto the first three components of sPLS-DA analysis of ImmuneSigDB singscore scores from TBI macrophages at 7dpi, 30dpi, and 8mpi, with 8mpi TBI microglia. (**f**) Heatmap of ImmuneSigDB singscore z-scores for significant terms for each component selected by sPLS-DA. The top terms for discriminating each group are shown. Individual animals are plotted (MDMs: 7dpi n=3 females, 3 males; 30dpi n=3 females, 3 males; 8mpi n=5 females, 6 males. Microglia 8mpi: n= 6 females, 6 males). Source data are provided as a Source Data file.

Supplementary Figure 8 - Ms4a3::Ai14dT RAWM

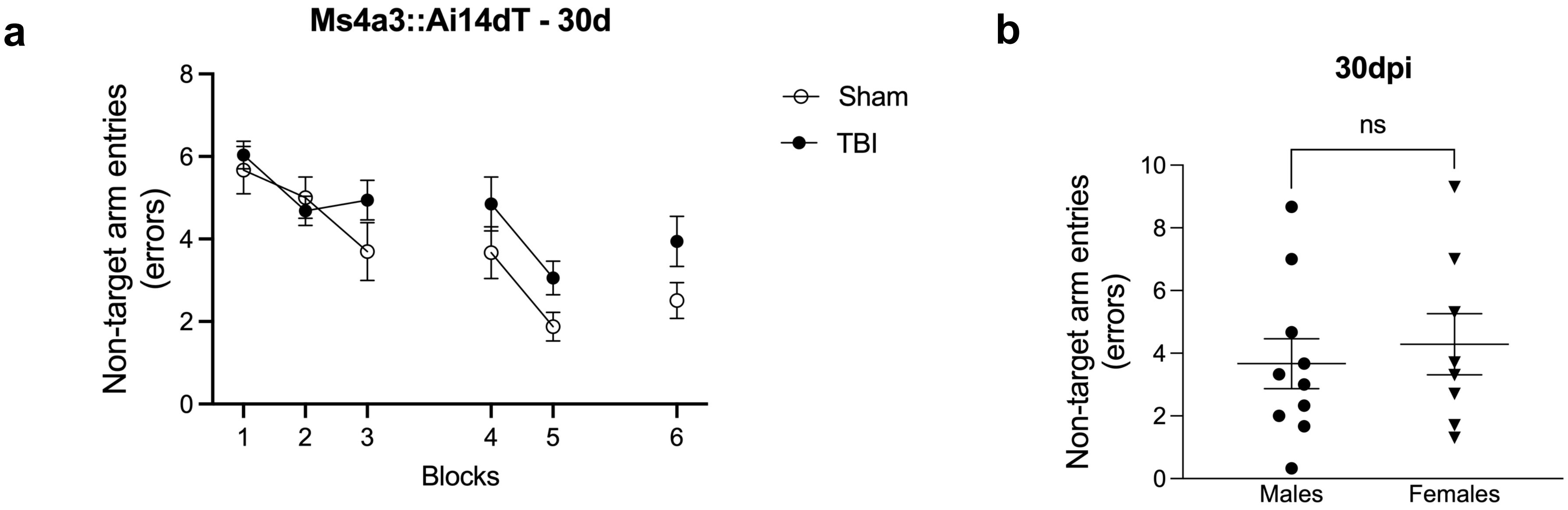

Source of Variation	% of total variation	P value	P value summary	Significant?	Geisser-Greenhouse's epsilon
Time x TBI	1.805	0.4649	ns	No	
Time	22.28	<0.0001	****	Yes	0.7333
TBI	3.395	0.0444	*	Yes	
Subject	20.61	0.0065	**	Yes	

Supplementary Figure 8 -
a) Ms4a3::Ai14D mice show learning and memory deficits in the RAWM 30 days after TBI. Data are expressed as mean of the examined variable ± SEM. **p*<0.05, ***p*<0.01, *****p*<0.001. Two way RM ANOVA. **b**) No sex differences were observed in the memory probe performance of TBI mice. Male mice are coded in circles and female in triangles. Individual animals are plotted (n = 6 males, 5 females (Sham) and 10 males, 8 females (TBI). Data are expressed as mean of the examined variable ± SEM. Unpaired t-test. Source data are provided as a Source Data file.

Supplementary Figure 9 - Infiltrated macrophages transcriptomes (Ccr2 and Ms4a3 combined) after brain engraftment mapped onto the Molecular Signatures and Reactome database

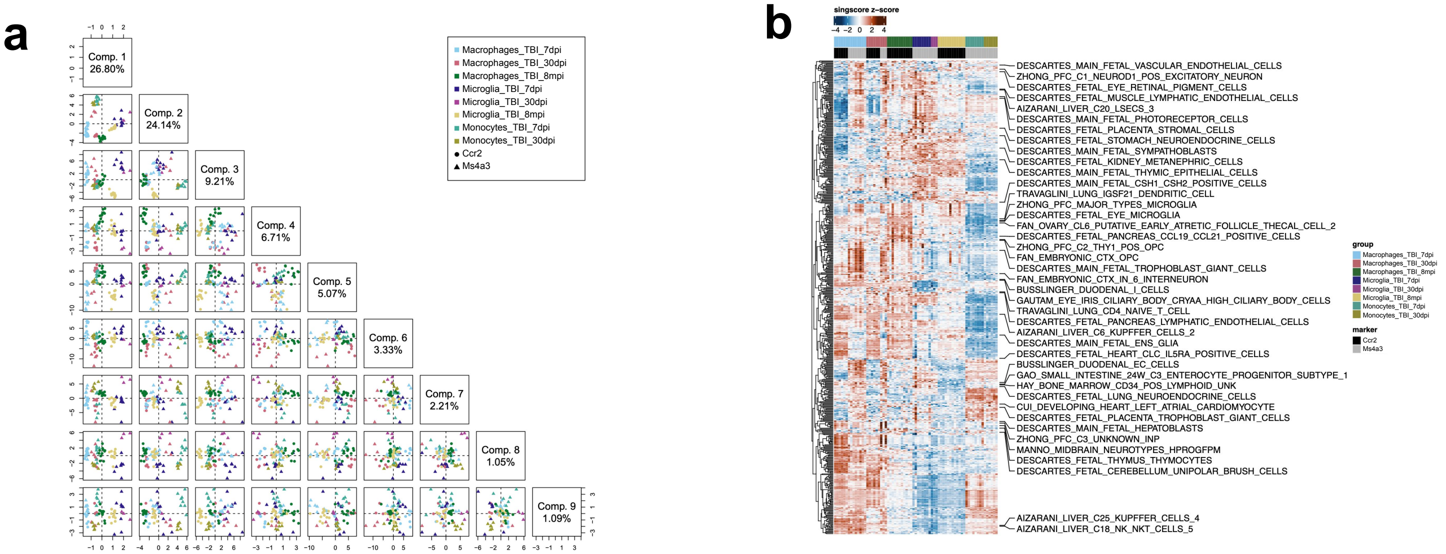

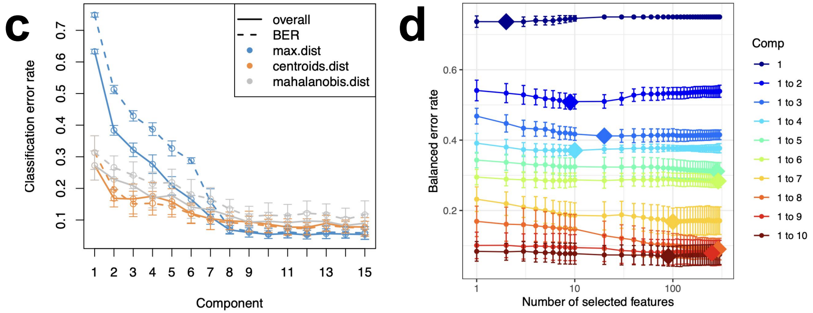

Supplementary Figure 9 - (**a**) Projections of samples onto the first 9 components of sPLS-DA analysis of MSigDB cell type signature singscore scores from TBI samples. (**b**) Heatmap of cell type signature singscore z-scores for significant terms for each component selected by sPLS-DA. The top terms for discriminating each group are shown. (**c**) Classification error rates per number of sPLS-DA components. 9 components were selected for the final models for both databases. (**d**) Balanced error rate per number of selected features per discriminant component. Source data are provided as a Source Data file.

Supplementary Figure 10 - overlap MDMs core signature with DIM/DAM published signatures

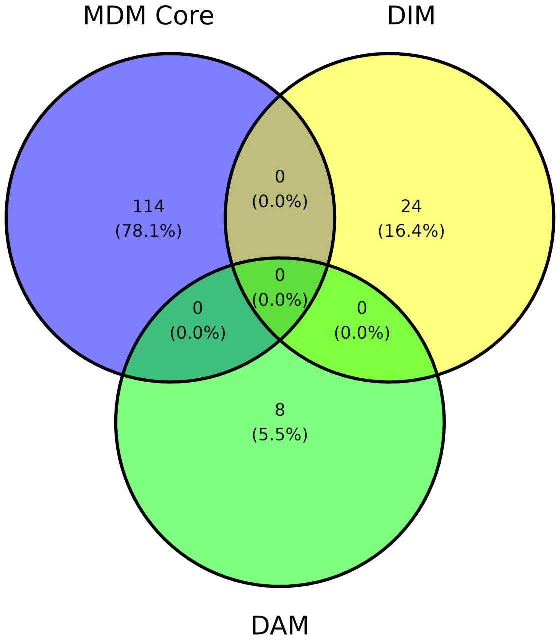

Supplementary Figure 10- Venn diagram of the MDM core signature with signatures of disease inflammatory microglia (DIMs) and disease-associated microglia (DAMs) collected from Silvin et al., (2023).

Supplementary Figure 11 - human TBI sn-RNA sequencing

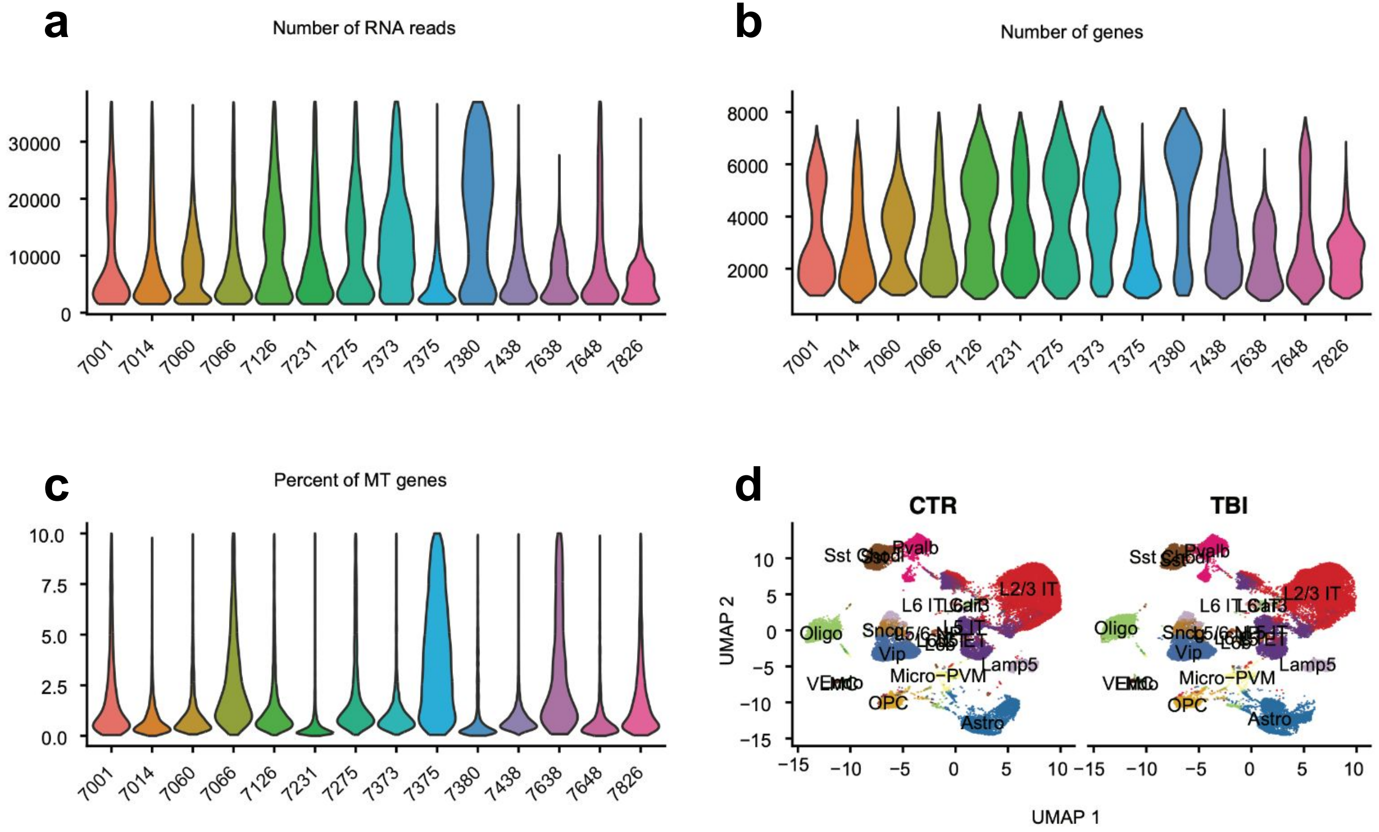

Supplementary Figure 11- Violin plots showing RNA quality metrics. RNA metrics include **(a)** the number of unique reads per cell, **(b)** the number of detected genes per cell, and **(c)** percentage of mitochondrial genes detected per cell. **(d)** Umap of snRNA-seq human dataset with level 2 annotations shown.

Supplementary Table S1- Demographics, TBI characteristics and pathology of the human tissue donors.

	Controls	TBI
<u>Demographics</u>		
Number	7	7
Age (SD)	47.9 (10.8)	47.7 (13.6)
% Male	100	100
% Contact sports history	14%	86%
<u>TBI data</u>		
Years post injury	--	18.4 (11.0)
% MVA	--	71%
% Fall from height	--	29%
% Repeated TBIs	--	42%
% LOC	--	100%
% Post-TBI seizures	--	14%
<u>Pathology</u>		
% Contusion (in OFC)	0%	43% (29%)
% + CTE	0%	57%
% ADRC intermediate	0%	0%
% + LBD	0%	14%
% + TDP-43	0%	0%

Supplementary Table S2 - Software and Algorithms

Software	Source
R (4.3.2)	R Core Team (2023). “R: A Language and Environment for Statistical Computing.” R Foundation for Statistical Computing, Vienna, Austria. <https://www.R-project.org/>.
nf-core/rnaseq pipeline (3.12.0)	10.5281/zenodo.1400710
tidyverse (2.0.0)	https://cran.r-project.org/web/packages/tidyverse/index.html
tidylog (1.1.0)	https://CRAN.R-project.org/package=tidylog
ComplexHeatmap (2.18.0)	https://bioconductor.org/packages/release/bioc/html/ComplexHeatmap.html
EnhancedVolcano (1.20.0)	https://bioconductor.org/packages/release/bioc/html/EnhancedVolcano.html
edgeR (4.0.16)	https://bioconductor.org/packages/release/bioc/html/edgeR.html
limma (3.58.1)	https://bioconductor.org/packages/release/bioc/html/limma.html
sva (3.50.0)	https://bioconductor.org/packages/release/bioc/html/sva.html
cqn (1.48.0)	https://bioconductor.org/packages/release/bioc/html/cqn.html
biomaRt (2.58.2)	https://www.bioconductor.org/packages/release/bioc/html/biomaRt.html
singscore (1.22.0)	https://bioconductor.org/packages/release/bioc/html/singscore.html
mixOmics (6.26.0)	https://www.bioconductor.org/packages/release/bioc/html/mixOmics.html
msigdbr (7.5.1)	https://igordot.github.io/msigdbr/
Cell Ranger (8.0.0)	https://www.10xgenomics.com/support/software/cell-ranger/latest/release-notes/cr-release-notes
Seurat (5.2.0)	https://cran.r-project.org/web/packages/Seurat/
PopsicleR (0.2.1)	https://github.com/bicciatolab/popsicleR
Azimuth (0.5.0)	https://satijalab.github.io/azimuth
UCell (2.7.7)	https://github.com/carmonalab/UCell
ShinyCell (2.1.0)	https://github.com/SGDDNB/ShinyCell
