# Supplementary material for: Fate mapping of peripherally-derived macrophages after traumatic brain injury in mice reveals a long-lasting population with a distinct transcriptomic signature
Source: Nat Commun. 2025 Oct 7;16:8898. doi: 10.1038/s41467-025-63952-8 (PMC12504527; doi:10.1038/s41467-025-63952-8)
Supplement: Supplementary file 2 — Description of Additional Supplementary Files [file 41467_2025_63952_MOESM2_ESM.pdf]

Description of Additional Supplementary Files

Supplementary Data 1: List of MDMs core signature genes, mouse and human orthologous
