# Supplementary material for: Fate mapping of peripherally-derived macrophages after traumatic brain injury in mice reveals a long-lasting population with a distinct transcriptomic signature
Source: Nat Commun. 2025 Oct 7;16:8898. doi: 10.1038/s41467-025-63952-8 (PMC12504527; doi:10.1038/s41467-025-63952-8)
Supplement: Supplementary file 4 — Reporting Summary [file 41467_2025_63952_MOESM4_ESM.pdf]

nature portfolio

Corresponding author(s): Susanna Rosi

Last updated by author(s): Aug 12, 2025

Reporting Summary

Nature Portfolio wishes to improve the reproducibility of the work that we publish. This form provides structure for consistency and transparency in reporting. For further information on Nature Portfolio policies, see our Editorial Policies and the Editorial Policy Checklist.

Statistics

For all statistical analyses, confirm that the following items are present in the figure legend, table legend, main text, or Methods section.

n/a	Confirmed	
☐	☒	The exact sample size (*n*) for each experimental group/condition, given as a discrete number and unit of measurement
☐	☒	A statement on whether measurements were taken from distinct samples or whether the same sample was measured repeatedly
☐	☒	The statistical test(s) used AND whether they are one- or two-sided<br>*Only common tests should be described solely by name; describe more complex techniques in the Methods section.*
☐	☒	A description of all covariates tested
☐	☒	A description of any assumptions or corrections, such as tests of normality and adjustment for multiple comparisons
☐	☒	A full description of the statistical parameters including central tendency (e.g. means) or other basic estimates (e.g. regression coefficient) AND variation (e.g. standard deviation) or associated estimates of uncertainty (e.g. confidence intervals)
☐	☒	For null hypothesis testing, the test statistic (e.g. *F*, *t*, *r*) with confidence intervals, effect sizes, degrees of freedom and *P* value noted<br>*Give P values as exact values whenever suitable.*
☒	☐	For Bayesian analysis, information on the choice of priors and Markov chain Monte Carlo settings
☒	☐	For hierarchical and complex designs, identification of the appropriate level for tests and full reporting of outcomes
☒	☐	Estimates of effect sizes (e.g. Cohen's *d*, Pearson's *r*), indicating how they were calculated

Our web collection on statistics for biologists contains articles on many of the points above.

Software and code

Policy information about availability of computer code

Data collection	Immunofluorescent images were acquired using Zen Blue (Zeiss), flow cytometry data were acquired using FACSDiva (BD), behavioral videos were acquired using Ethovision XT (Noldus Information Technology).
Data analysis	FlowJo (BD), Imaris (Bitplane), MATLAB (MathWorks), Prism (Graphpad). RNA-Seq data was processed using the nf-core/rnaseq pipeline (v3.12.0) and downstream analyses were performed in R (v4.3.2). Essential R packages include tidyverse (v2.0.0), tidylog (v1.1.0), ComplexHeatmap (v2.18.0), edgeR (v4.0.16), limma (v3.58.1), sva (v3.50.0), cqn (1.48.0), biomaRt (v2.58.2), singscore (v1.22.0), mixOmics (6.26.0), and msigdbr (7.5.1), Cell Ranger (8.0.0), Seurat (5.2.0), PopsicleR (0.2.1), Azimuth (0.5.0), UCell (2.7.7), ShinyCell (2.1.0)

For manuscripts utilizing custom algorithms or software that are central to the research but not yet described in published literature, software must be made available to editors and reviewers. We strongly encourage code deposition in a community repository (e.g. GitHub). See the Nature Portfolio guidelines for submitting code & software for further information.

Data

Policy information about availability of data

All manuscripts must include a data availability statement. This statement should provide the following information, where applicable:

- Accession codes, unique identifiers, or web links for publicly available datasets
- A description of any restrictions on data availability
- For clinical datasets or third party data, please ensure that the statement adheres to our policy

The raw files for the datasets generated in this manuscript have been deposited in the National Center for Biotechnology Information Gene Expression Omnibus (GEO). Bulk RNA-seq libraries of CD11b+ CD45+ cells from whole brains of Ccr2-creERT2::Ai14D (7, 30 days after TBI) have been deposited under accession code: GSE283558 [https://www.ncbi.nlm.nih.gov/geo/query/acc.cgi?acc=GSE283558]. Bulk RNA-seq libraries of CD11b+ CD45+ cells from whole brains of Ccr2-creERT2::Ai14D (8 months after TBI) have been deposited under accession code: GSE283556 [https://www.ncbi.nlm.nih.gov/geo/query/acc.cgi?acc=GSE283556]. Bulk RNA-seq libraries of CD11b+ CD45+ cells from whole brains of Ms4a3-cre::Ai14D have been deposited under accession code: GSE283560 [https://www.ncbi.nlm.nih.gov/geo/query/acc.cgi?acc=GSE283560]. The single-cell RNA-seq libraries of human TBI samples have been deposited under accession code: GSE294775 [https://www.ncbi.nlm.nih.gov/geo/query/acc.cgi?&acc=GSE294775]. An interactive shiny app for visualizing the TBI datasets is available here: https://altoslabs.shinyapps.io/tbi-human-atlas/. The Seattle Alzheimer's Disease Brain Cell Atlas is publicly available and was downloaded from: https://portal.brain-map.org/explore/seattle-alzheimers-disease/seattle-alzheimers-disease-brain-cell-atlas-download?edit&language=en.
All data supporting the findings of this study are available within the paper and its Supplementary Information. Source data are provided with this paper.

Research involving human participants, their data, or biological material

Policy information about studies with human participants or human data. See also policy information about sex, gender (identity/presentation), and sexual orientation and race, ethnicity and racism.

Reporting on sex and gender	Informations on donors demographic are in Supplementary Table S2. 100% of the donors for this study were male. No information on gender was provided by the Pacific Northwest Brain Donor Network.
Reporting on race, ethnicity, or other socially relevant groupings	No information on race/ethnicity or other socially relevant grouping was provided to us by the Pacific Northwest Brain Donor Network. All the available Informations on donors demographic are in Supplementary Table S2.
Population characteristics	All the available Informations on donors demographic are in Supplementary Table S2. Age (SD) of the Controls is 47.9 (10.8) and of TBI is 47.7 (13.6)
Recruitment	Brain donors in this study were part of the Pacific Northwest Brain Donor Network, which collects tissue from military veterans and civilians, both with and without a history of traumatic brain injury in collaboration with local Seattle area medical examiners. Consent for brain donation was obtained from the legal next of kin who agreed to be contacted and were interested in the UW research program.
Ethics oversight	Brain donation studies at the University of Washington (UW) were approved by the UW School of Medicine Compliance office and Institutional Review Board and all brain donation was collected with informed consent. Brain donors in this study were part of the Pacific Northwest Brain Donor Network, which collects tissue from military veterans and civilians, both with and without a history of traumatic brain injury in collaboration with local Seattle area medical examiners. Consent for brain donation was obtained from the legal next of kin who agreed to be contacted and were interested in the UW research program.

Note that full information on the approval of the study protocol must also be provided in the manuscript.

Field-specific reporting

Please select the one below that is the best fit for your research. If you are not sure, read the appropriate sections before making your selection.

[x] Life sciences [ ] Behavioural & social sciences [ ] Ecological, evolutionary & environmental sciences

For a reference copy of the document with all sections, see nature.com/documents/nr-reporting-summary-flat.pdf

Life sciences study design

All studies must disclose on these points even when the disclosure is negative.

Sample size	Sample size calculations were based on previously published data. No statistical methods were used to predetermine sample size. Figure legends indicate the sample sizes for each set of experiments.
Data exclusions	Only animals that fully recovered from the surgical procedures as exhibited by normal behavior, healed sutures, and weight maintenance monitored throughout the duration of the experiments were used. For downstream analysis, group outliers were determined (ROUT method, Q=1%) and excluded.
Replication	Biological replicates are detailed in the figure legends and are plotted as individual animals/samples when appropriate. RNA-Seq technical

	replicates were processed independently then summed per biological replicate before downstream analyses.
Randomization	Animals were randomly assigned to experimental groups
Blinding	All analysis performed blinded by experimental group

Reporting for specific materials, systems and methods

We require information from authors about some types of materials, experimental systems and methods used in many studies. Here, indicate whether each material, system or method listed is relevant to your study. If you are not sure if a list item applies to your research, read the appropriate section before selecting a response.

Materials & experimental systems

n/a	Involved in the study
☐	☒ Antibodies
☒	☐ Eukaryotic cell lines
☒	☐ Palaeontology and archaeology
☐	☒ Animals and other organisms
☒	☐ Clinical data
☒	☐ Dual use research of concern
☒	☐ Plants

Methods

n/a	Involved in the study
☒	☐ ChIP-seq
☐	☒ Flow cytometry
☒	☐ MRI-based neuroimaging

Antibodies

Antibodies used	For flow cytometry analysis, blood and brain samples were blocked with CD16/32 Fc block (BD Biosciences #553141) and stained with fluorophore-conjugated antibodies: CD11b-AF700 (BD Pharmingen, #557690), CD11b-BV421 (BD Pharmingen, #562605), CD45-FITC (BD Pharmingen, #553080), CD45-APC-Cy7 (BioLegend, #304014), Ly6C-V450 (BD Pharmingen, #560594), Ly6C-APC (BD Pharmingen, #560600) Ly6G-PE-Cy7 (BD Pharmingen,# 560601) and Ly6G-BV711 (BD Pharmingen, #563979). For immunofluorescence analysis, samples were stained stained with 488-conjugated rabbit anti-Iba1, Cell Signaling, #20825, 1:100 and anti-P2yr12, Anaspec, #AS-55043A, 1:400 overnight at 4C and then 2h at room temperature with donkey anti-rabbit AF647, Invitrogen, #A32795, 1:400. Nuclei were stained with DAPI. For the in vivo phagocytosis assay, nuclei were stained with H3K9me3 antibody (abcam, #ab176916, 1:2000) overnight at 4C and then 2h at room temperature with donkey anti-goat AF488, Invitrogen #A11055, 1:400, as the blue channel was used by the latex beads.
Validation	All the antibodies were used following manufacturer's instructions and have been validated by the vendors and previous studies performed by our laboratory or by others.

Animals and other research organisms

Policy information about studies involving animals; ARRIVE guidelines recommended for reporting animal research, and Sex and Gender in Research

Laboratory animals	Ccr2-creERT2-mKate2 mice were obtained from the University of Zurich (UZH). Ms4a3-cre were obtained from the Singapore Immunology Network (SIgN), A*STAR. Ai14D reporter mice were purchased from Jackson laboratory (#007914). Ccr2-creERT2-mKate2 and Ms4a3-cre mice were crossed with Ai14D mice to obtain Ccr2-creERT2::Ai14D and Ms4a3-cre::Ai14D mice. Mice were group housed (by sexes and injury state) in environmentally controlled conditions with a reverse light cycle (12:12 h light: dark cycle at 21 ± 1 ◦C; ~50% humidity) and provided food and water ad libitum.
Wild animals	No wild animals were used for this study.
Reporting on sex	Both female and male mice were used for this study.
Field-collected samples	The study did not involve data collected from the field.
Ethics oversight	All experiments were conducted in accordance with National Institutes of Health (NIH) Guide for the Care and Use of Laboratory Animals and approved by the Institutional Animal Care and Use Committee (IACUC) of both the University of California, San Francisco (AN184326) and from Altos Labs, Inc (EB22-101-100) to ensure compliance with ethical standards and the humane treatment of animals.

Note that full information on the approval of the study protocol must also be provided in the manuscript.

Plants

Seed stocks

Report on the source of all seed stocks or other plant material used. If applicable, state the seed stock centre and catalogue number. If plant specimens were collected from the field, describe the collection location, date and sampling procedures.

Novel plant genotypes

Describe the methods by which all novel plant genotypes were produced. This includes those generated by transgenic approaches, gene editing, chemical/radiation-based mutagenesis and hybridization. For transgenic lines, describe the transformation method, the number of independent lines analyzed and the generation upon which experiments were performed. For gene-edited lines, describe the editor used, the endogenous sequence targeted for editing, the targeting guide RNA sequence (if applicable) and how the editor was applied.

Authentication

Describe any authentication procedures for each seed stock used or novel genotype generated. Describe any experiments used to assess the effect of a mutation and, where applicable, how potential secondary effects (e.g. second site T-DNA insertions, mosiacism, off-target gene editing) were examined.

Flow Cytometry

Plots

Confirm that:

☒ The axis labels state the marker and fluorochrome used (e.g. CD4-FITC).

☒ The axis scales are clearly visible. Include numbers along axes only for bottom left plot of group (a 'group' is an analysis of identical markers).

☒ All plots are contour plots with outliers or pseudocolor plots.

☒ A numerical value for number of cells or percentage (with statistics) is provided.

Methodology

Sample preparation

Blood: Red blood cells were lysate using RBC lysis buffer (BioLegend, #420301) and samples were then blocked with CD16/32 Fc block (BD Biosciences #553141) and stained with fluorophore-conjugated antibodies: CD11b-AF700 (BD Pharmingen, #557690), CD11b-BV421 (BD Pharmingen, #562605), CD45-FITC (BD Pharmingen, #553080), CD45-APC-Cy7 (BioLegend, #304014), Ly6C-V450 (BD Pharmingen, #560594), Ly6C-APC (BD Pharmingen, #560600) Ly6G-PE-Cy7 (BD Pharmingen,# 560601) and Ly6G-BV711 (BD Pharmingen, #563979). Cells were then washed in FACS buffer (1×DPBS with 0.5% BSA fraction V and 2% FBS) and used for analyses.
Brain: Mice were euthanized and perfused with cold PBS. Brains were immediately removed and dissociated using a Neural Tissue Dissociation kit (P) (Miltenyi Biotec, #130-092-628). Cells were then resuspended in 30% Percoll solution diluted in RPMI medium, and centrifuged at 800 g for 20 min at 4 °C. Cell pellets were washed with FACS buffer (1×DPBS with 0.5% BSA fraction V and 2% FBS or Rockland, #MB-086-0500), blocked with mouse CD16/32 Fc block (BD Biosciences, #553141) and then stained with fluorophore-conjugated antibodies: CD11b-AF700 (BD Pharmingen, #557690), CD11b-BV421 (BD Pharmingen, #562605), CD45-FITC (BD Pharmingen, #553080), CD45-APC-Cy7 (BioLegend, #304014), Ly6C-V450 (BD Pharmingen, #560594), Ly6C-APC (BD Pharmingen, #560600) Ly6G-PE-Cy7 (BD Pharmingen,# 560601) and Ly6G-BV711 (BD Pharmingen, #563979). Cells were then washed in FACS buffer and used for analyses or sorted.

Instrument

Data were collected on BD FACSymphony™ A3 Cell Analyzer and on BD FACSAriaTM (III and Fusion) cell sorters (BD Biosciences, V8.0.1)

Software

FACSDiva (BD)

Cell population abundance

Cell population abundance is displayed in the relevant bar plots as a percentage.

Gating strategy

Cells were gated using FSC/SSC plot, and singlets were gated using SSC-A/FSC-W plot. For blood, inflammatory monocytes (tdTomato+) were gated as CD45+, CD11b+, Ly6G-,Ly6C high, tdTomato+; patrolling monocytes were gated as CD45+, CD11b +, Ly6G negative and Ly6C low; neutrophils were gated as CD45+, CD11b+, Ly6C+, Ly6G+. For brain samples, microglia were gated as CD11b+, CD45 mid/low, tdTomato-: monocyte-derived macrophages were gated as CD11b+, CD45+, tdTomato+.

☒ Tick this box to confirm that a figure exemplifying the gating strategy is provided in the Supplementary Information.
